# Perfluoroalkyl substances and time to pregnancy in couples from Greenland, Poland and Ukraine

**DOI:** 10.1186/1476-069X-13-116

**Published:** 2014-12-22

**Authors:** Kristian T Jørgensen, Ina O Specht, Virissa Lenters, Cathrine C Bach, Lars Rylander, Bo AG Jönsson, Christian H Lindh, Aleksander Giwercman, Dick Heederik, Gunnar Toft, Jens Peter Bonde

**Affiliations:** Department of Occupational and Environmental Medicine, Bispebjerg Hospital, Copenhagen University Hospital, Copenhagen, Denmark; Division of Environmental Epidemiology, Institute for Risk Assessment Sciences, Utrecht University, Utrecht, The Netherlands; Perinatal Epidemiology Research Unit, Aarhus University Hospital, Skejby, Denmark; Division of Occupational and Environmental Medicine, Lund University, Lund, Sweden; Reproductive Medicine Centre, Malmö University Hospital, Lund University, Malmö, Sweden; Department of Occupational Medicine, Danish Ramazzini Center, Aarhus University Hospital, Aarhus, Denmark

**Keywords:** Fecundity, Perfluorinated compounds, Infertility, Persistent environmental pollutants

## Abstract

**Background:**

Perfluoroalkyl substances (PFAS) are suggested to affect human fecundity through longer time to pregnancy (TTP). We studied the relationship between four abundant PFAS and TTP in pregnant women from Greenland, Poland and Ukraine representing varying PFAS exposures and pregnancy planning behaviors.

**Methods:**

We measured serum levels of perfluorooctanoic acid (PFOA), perfluorooctane sulfonate (PFOS), perfluorohexane sulfonic acid (PFHxS) and perfluorononanoic acid (PFNA) in 938 women from Greenland (448 women), Poland (203 women) and Ukraine (287 women). PFAS exposure was assessed on a continuous logarithm transformed scale and in country-specific tertiles. We used Cox discrete-time models and logistic regression to estimate fecundability ratios (FRs) and infertility (TTP >13 months) odds ratios (ORs), respectively, and 95% confidence intervals (CI) according to PFAS levels. Adjusted analyses of the association between PFAS and TTP were done for each study population and in a pooled sample.

**Results:**

Higher PFNA levels were associated with longer TTP in the pooled sample (log-scale FR = 0.80; 95% CI 0.69-0.94) and specifically in women from Greenland (log-scale FR = 0.72; 95% CI 0.58-0.89). ORs for infertility were also increased in the pooled sample (log-scale OR = 1.53; 95% CI 1.08-2.15) and in women from Greenland (log-scale OR = 1.97; 95% CI 1.22-3.19). However, in a sensitivity analysis of primiparous women these associations could not be replicated. Associations with PFNA were weaker for women from Poland and Ukraine. PFOS, PFOA and PFHxS were not consistently associated with TTP.

**Conclusions:**

Findings do not provide consistent evidence that environmental exposure to PFAS is impairing female fecundity by delaying time taken to conceive.

**Electronic supplementary material:**

The online version of this article (doi:10.1186/1476-069X-13-116) contains supplementary material, which is available to authorized users.

Perfluoroalkyl substances (PFAS) (also referred to as perfluorinated compounds) are used in a wide range of industrial and consumer products. These man-made contaminants are biopersistent with half-lives reported from three to nine years and have been detected in many human populations [1, 2]. The main route of human exposure is from intake of contaminated food and drinking water [3, 4] However, house dust may also be contributing to PFAS exposure [5]. Although some PFAS, like perfluorooctane sulfonate (PFOS), have been phased out since 2000, the compounds that already exist in the environment may potentially continue to affect human health for many years to come because of their high persistency [6]. Furthermore, new PFAS have been introduced and their possible impact on human health has not been thoroughly investigated.

Toxicological effects of PFAS have mainly been studied in experimental animal models and include reproductive impairments [7–9]. Health effects in humans are not well-established [10, 11]. Four previous studies have addressed the association between PFAS and couple fecundity in humans measured by time to pregnancy (TTP). A study from Denmark examined PFOS and perfluorooctanoic acid (PFOA) exposure levels in blood samples of pregnant women and found reduced fecundability ratios and increased infertility in women with PFOS and PFOA levels above the 25th percentile of the exposure distribution corresponding to plasma concentrations of 26.6 ng/ml and 4.9 ng/ml, respectively [12]. These associations were confirmed when the authors restricted the analysis to pregnant primiparous women [13]. A case–control study from Norway found increased odds of subfecundity (TTP >12 months) in women with high exposure to PFOS and PFOA. However, these findings were not confirmed when restricting to primiparous women [14]. A prospective study from the US assessed the relationship between seven PFAS as well as other persistent environmental pollutants and couple fecundity in pregnancy planners. Overall they did not find much support for an association between PFAS exposure and TTP [15]. Also, a Danish prospective study of pregnancy planners found no association between seven PFAS and TTP despite relatively high exposure levels [16].

On the basis of the existing literature it is therefore unresolved whether environmental exposure to PFAS may affect human fecundity. This study is addressing the association between exposure to PFAS and time taken to conceive and is distinguished from earlier studies by inclusion of three study populations from three geographical regions which provides opportunities to examine consistency of findings across different populations studied according to uniform study protocols. Moreover, this is the first study taking PFAS exposure of both partners into account. The study included measurements of the four PFAS found in highest concentrations in humans; PFOA, PFOS, perfluorohexane sulfonic acid (PFHxS) and perfluorononanoic acid (PFNA) [10].

## Methods

### Study populations

This study is based on the INUENDO cohort including a total of 1710 pregnant women from Greenland, Poland (Warsaw) and Ukraine (Kharkiv) enrolled during antenatal care visits between May 2002 and February 2004. The participants were at least 18 years old and born in the country of study. They were asked to complete a questionnaire and give a blood sample at enrolment. The participation rates were 90% (598/665) in Greenland, 68% (472/690) in Poland, and 26% (640/2478) in Ukraine. One of the reasons that a large number of pregnant women refused to participate in Ukraine was concern that the collection of a blood sample (altogether 35 ml whole blood) would imply a health risk [17]. An analysis of demographic and reproductive data in a large sample of women that declined participation did not indicate that the Ukranian sample is a select group [17]. The INUENDO study population has been described in detail elsewhere [17].

A group of 437 women became pregnant despite use of birth control and thus had no eligible TTP, and for 131 women we had no information regarding TTP. A further 204 women did not provide a blood sample. Thus, the final cohort of women providing both questionnaire data and a blood sample comprised 938 women with 448, 203 and 287 women from Greenland, Poland and Ukraine, respectively.

### Time to pregnancy data

Information on the time taken to conceive the current pregnancy was obtained by the following two questions: (1) Leading up to this pregnancy, when was it that you started having sexual intercourse without using any birth control to prevent pregnancy?" Month:______ Year:______. We now call this the "STARTING TIME". (2) How long was it from that "STARTING TIME" until you became pregnant? (The date you became pregnant is the date you conceived) How long? Weeks:______ and/or Months:______ and/or Years: ______. Further details are provided in [17].

### Analysis of PFAS

The laboratory analysis of serum concentrations of PFOA, PFOS, PFHxS and PFNA has been described in detail elsewhere [18, 19]. In short, PFAS were analyzed with liquid chromatography tandem mass spectrometry. Limits of detection (LOD) were the following: PFOA 0.04 ng/ml, PFOS 0.2 ng/ml, PFHxS 0.02 ng/ml and PFNA 0.03 ng/ml. For all four PFAS the detection frequency was 100%. The reproducibility of the method was determined as the coefficient of variation of duplicate samples analyzed on different days and found to be 6% for PFOA, 5% for PFOS, 8% for PFHxS and 9% for PFNA. The analyses of PFOS and PFOA are part of the Round Robin inter-comparison program (Professor Dr. med. Hans Drexler, Institute and Out-Patient Clinic for Occupational, Social and Environmental Medicine, University of Erlangen-Nuremberg, Germany) with results within the tolerance limits.

### Statistical analyses

Each study population was studied separately where all measures of female serum concentrations of PFAS were i) categorized into country-specific tertiles with the lowest category for each country serving as reference category, and ii) handled on a continuous logarithm transformed scale. We also studied the association between TTP and PFAS on the continuous log-scale for a pooled sample consisting of all 938 study participants. We calculated fecundability ratios (FRs) with 95% confidence intervals (CIs) using a discrete-time Cox regression model that handled the underlying time scale – the TTP in months – as a discrete scale. The FR represents the probability of conceiving during a time period (e.g., one month or one menstrual cycle) within one group compared to the probability in the reference group. Instead of censoring after 12 months, TTP was censored after 13 months in order to take digit preference into account (proportionally more women recall TTPs of 12 months compared to 11 and 13 months). All FR analyses were adjusted for maternal age (<25, 25–29, 30+ years), self-reported smoking status (yes, no), parity status (primiparous, 1, 2+ children), maternal body mass index (BMI, <20, 20–24, 25+) and gestational week at interview and blood sampling (gestational week 1–20, 21–30, 31+). In the pooled analysis an additional adjustment was made for country. Odds ratios (ORs) and 95% CIs were calculated in a logistic regression analysis in which women with TTPs >13 months were categorized as infertile (despite they eventually became pregnant), while women with TTPs ≤13 months were fertile. The OR analysis was adjusted for the same covariates as in the Cox regression model. Spearman correlation coefficients (r_S_) were used to assess country-specific correlations between PFAS in women and correlations between levels in men (see below) and women. Two-sided statistical tests were applied and P-values <0.05 and 95% CIs excluding unity were considered statistically significant. The statistical analysis was conducted in SAS statistical software version 9.3.

In a number of supplementary analyses we tested the robustness of the main findings. I) We restricted to primiparous women and calculated FRs and infertility ORs based on the original PFAS tertile levels used in the main analysis. Restriction to primiparous women was considered essential to bypass reverse causality that may be introduced by the association between prolonged time to pregnancy and increasing PFAS levels with time after delivery [20, 21] II) We analyzed the association between male serum levels of PFAS and couple TTP for 401 male spouses of the female participants who also provided a blood sample (160 men from Greenland, 146 men from Poland, and 95 men from Ukraine). The analysis concerning male PFAS levels was adjusted for paternal age (<25, 25–29, 30+ years), paternal BMI (<20, 20–24, 25+) and maternal age (<25, 25–29, 30+ years). In the pooled sample we additionally adjusted for country. FRs were based on couple TTP according to country-specific PFAS tertiles and the continuous log-scale of PFAS exposure.

### Ethical approval

The study was approved by the relevant scientific ethical committees in Greenland, Poland and Ukraine and all participants signed an informed consent.

## Results

The median TTP was 4 [interquartile range (IQR) 2–8], 4 (IQR 2–9) and 5 (IQR 3–13) months for women from Greenland, Poland and Ukraine, respectively. The proportion of infertile couples (TTP >13 months) was 14% in Greenland, 16% in Poland and 20% in Ukraine. The median gestational week of enrolment and blood sampling was week 24 (IQR 18–32) for Greenland, 33 (IQR 31–35) for Poland, and 23 (IQR 12–33) for Ukraine. Women from Greenland generally had higher BMI, more children and were more likely to smoke compared to women from Poland and Ukraine. The educational level was higher for women from Poland than for women from Greenland and Ukraine. Women from Ukraine were generally younger than women from Greenland and Poland (Table 1). The coefficient of correlation for parental ages was ranging from 0.56 (95% CI 0.49-0.62) in Poland to 0.69 (95% CI 0.65-0.73) in Ukrania.

**Table 1:** **Characteristics of the cohort according to body mass index, age, number of previous childbirths, smoking status, education and gestational week of blood sampling**

	Greenland	Poland	Ukraine
Maternal BMI	n (%)	n (%)	n (%)
<20	56 (13)	61 (30)	93 (32)
20-24	224 (50)	122 (60)	156 (54)
25+	164 (37)	17 (8)	37 (13)
Missing	4 (1)	3 (1)	1 (0)
Maternal age (yrs)			
<25	224 (50)	28 (14)	200 (70)
25-29	93 (21)	134 (66)	61 (21)
30+	117 (26)	41 (20)	22 (8)
Missing	14 (3)	0 (0)	4 (1)
Previous childbirths			
0	138 (31)	187 (92)	227 (79)
1	138 (31)	12 (6)	52 (18)
2+	169 (38)	1 (0)	4 (1)
Missing	3 (1)	3 (1)	4 (1)
Smoking status			
Non-smoker	118 (26)	167 (82)	222 (77)
Smoker	330 (74)	36 (18)	65 (23)
Maternal Education			
Left school at age ≤17	187 (42)	0 (0)	87 (30)
Left school at age ≥18	44 (10)	6 (3)	77 (27)
Post school training	162 (36)	18 (9)	90 (31)
University degree	7 (1)	157 (77)	0 (0)
Other or unknown	48 (11)	22 (11)	33 (12)
Time from last menstrual bleeding to interview and blood sampling (weeks)			
01-20	141 (31)	1 (0)	112 (39)
21-30	151 (34)	35 (17)	61 (21)
31-42	128 (29)	152 (75)	89 (31)
Missing	28 (6)	15 (7)	25 (9)

### Concentrations of PFAS in women from Greenland, Poland and Ukraine

The median levels of serum PFAS in women from Greenland, Poland and Ukraine are presented in Table 2.

**Table 2:** **Overall and country-specific median serum levels of PFAS (ng/ml) in women from Greenland, Poland and Ukraine and exposure ranges in the country specific tertiles**

	Overall	Greenland	Poland	Ukraine
Perfluorooctanoic acid (PFOA)	1.65	1.83	2.67	0.92
Low		0.50-1.51	0.97-2.16	0.24-0.78
Medium		1.51-2.21	2.16-3.10	0.78-1.10
High		2.22-5.13	3.13-9.84	1.11-9.79
Perfluorooctane sulfonic acid (PFOS)	10.60	20.32	7.97	4.93
Low		4.10-17.15	2.53-6.97	0.75-3.97
Medium		17.17-24.02	6.98-9.27	3.98-5.67
High		24.04-87.27	9.36-21.28	5.67-18.09
Perfluorohexane sulfonic acid (PFHxS)	1.94	2.04	2.35	1.55
Low		0.39-1.64	0.79-1.83	0.22-1.14
Medium		1.64-2.60	1.84-2.98	1.17-1.98
High		2.60-13.79	3.00-10.82	1.99-19.71
Perfluorononanoic acid (PFNA)	0.64	0.70	0.60	0.60
Low		0.20-0.56	0.19-0.48	0.14-0.48
Medium		0.56-0.90	0.48-0.73	0.48-0.73
High		0.90-5.71	0.74-2.76	0.73-3.72

PFAS perfluoroalkyl substances.

### Correlations between PFAS

PFOS was correlated with PFOA (r_S_ = 0.50) and PFNA (r_S_ = 0.74) in women from Greenland. In women from Poland, PFOS was correlated with PFOA (r_S_ = 0.52) and PFNA (r_S_ = 0.50) while PFOA and PFNA also were correlated (r_S_ = 0.69). Similar correlations were found for women from Ukraine (PFOS and PFOA, r_S_ = 0.52; PFOS and PFNA, r_S_ = 0.55; PFOA and PFNA, r_S_ = 0.52). PFHxS was to a lesser extent correlated with the other PFAS (from r_S_ = 0.16 for PFHxS and PFNA in Ukraine to r_S_ = 0.38 for PFHxS and PFOS in Greenland). We also assessed correlations of PFAS in 152, 145 and 86 couples from Greenland, Poland and Ukraine, respectively, for whom we had a blood sample available from both the man and woman. In Greenland PFNA levels in men and women were correlated (r_S_ = 0.52), PFOS (r_S_ = 0.22) and PFHxS (r_S_ = 0.22) were weakly correlated, while PFOA levels were not (r_S_ = 0.06). In Poland PFNA levels in men and women were also correlated (r_S_ = 0.48), while the other PFAS were less strongly correlated (PFOA r_S_ = 0.29, PFOS r_S_ = 0.25, and PFHxS r_S_ = 0.14). In Ukraine PFAS levels in men and women were generally not correlated (PFOA r_S_ = 0.20; PFOS r_S_ = -0.09; PFHxS r_S_ = 0.13; PFNA r_S_ =0.14).

### PFAS and fecundability and infertility ratios

#### PFOA

PFOA was not associated with FRs (Table 3) or infertility ORs (Table 4) in women specifically from Greenland, Poland and Ukraine or in the pooled sample (continuous log-scale FR = 1.04, 95% CI 0.87-1.25 and OR = 1.11, 95% CI 0.74-1.66).

**Table 3:** **Fecundability ratios according to female serum concentrations of PFAS**

		PFOA	PFOS	PFHxS	PFNA
Greenland	N	FR (95% CI)	FR (95% CI)	FR (95% CI)	FR (95% CI)
Low	150	1 (Reference)	1 (Reference)	1 (Reference)	1 (Reference)
Medium	149	0.95 (0.72, 1.26)	0.80 (0.60, 1.05)	1.05 (0.79, 1.38)	0.76 (0.58, 1.00)
High	149	0.87 (0.65, 1.18)	0.80 (0.60, 1.05)	0.90 (0.68, 1.19)	0.71 (0.54, 0.94)
Continuous log-scale	448	0.97 (0.73, 1.27)	0.83 (0.64, 1.07)	0.97 (0.78, 1.20)	0.72 (0.58, 0.89)
Poland					
Low	68	1 (Reference)	1 (Reference)	1 (Reference)	1 (Reference)
Medium	68	0.94 (0.62, 1.42)	0.87 (0.57, 1.33)	0.86 (0.57, 1.30)	0.94 (0.62, 1.41)
High	67	1.08 (0.71, 1.64)	0.90 (0.60, 1.37)	0.94 (0.62, 1.42)	1.31 (0.87, 1.98)
Continuous log-scale	203	1.21 (0.79, 1.84)	0.94 (0.58, 1.50)	0.90 (0.65, 1.24)	1.35 (0.93, 1.95)
Ukraine					
Low	96	1 (Reference)	1 (Reference)	1 (Reference)	1 (Reference)
Medium	96	1.12 (0.78, 1.59)	0.89 (0.62, 1.27)	0.85 (0.59, 1.23)	0.79 (0.55, 1.12)
High	95	1.04 (0.72, 1.51)	0.93 (0.64, 1.34)	1.11 (0.78, 1.58)	0.88 (0.62, 1.26)
Continuous log-scale	287	1.20 (0.87, 1.67)	1.06 (0.78, 1.45)	1.05 (0.84, 1.31)	0.74 (0.53, 1.04)
Pooled sample					
Continuous log-scale	938	1.04 (0.87, 1.25)	0.90 (0.76, 1.07)	0.97 (0.85, 1.11)	0.80 (0.69, 0.94)

FRs are presented in country-specific tertiles and according to a continuous logarithm-transformed scale.

CI confidence intervals, FR fecundability ratio, N number of women, PFAS perfluoroalkyl substances.

The FR analyses are adjusted for parity, gestational week of blood sampling, smoking status, maternal age and BMI. In addition the pooled analysis is adjusted for country.

**Table 4:** **Infertility odds ratios according to female serum concentrations of PFAS**

	N		PFOA		PFOS	PFHxS			PFNA
Greenland		n	OR (95% CI)	n	OR (95% CI)	n	OR (95% CI)	n	OR (95% CI)
Low	150	18	1 (Reference)	14	1 (Reference)	20	1 (Reference)	12	1 (Reference)
Medium	149	22	1.30 (0.66, 2.54)	23	1.73 (0.85, 3.49)	18	0.90 (0.45, 1.76)	23	1.95 (0.95, 3.99)
High	149	22	1.57 (0.76, 3.25)	25	1.82 (0.90, 3.67)	24	1.22 (0.64, 2.36)	27	2.15 (1.05, 4.42)
Continuous log-scale	448	62	1.20 (0.61, 2.37)	62	1.74 (0.97, 3.13)	62	0.99 (0.59, 1.65)	62	1.97 (1.22, 3.19)
Poland									
Low	68	10	1 (Reference)	7	1 (Reference)	10	1 (Reference)	13	1 (Reference)
Medium	68	12	1.25 (0.50, 3.13)	13	1.93 (0.72, 5.18)	13	1.52 (0.60, 3.84)	11	0.92 (0.38, 2.22)
High	67	11	1.41 (0.55, 3.61)	13	1.92 (0.72, 5.14)	10	1.07 (0.41, 2.80)	9	0.65 (0.26, 1.66)
Continuous log-scale	203	33	1.11 (0.44, 2.80)	33	2.06 (0.69, 6.12)	33	1.05 (0.50, 2.20)	33	0.74 (0.32, 1.72)
Ukraine									
Low	96	19	1 (Reference)	18	1 (Reference)	17	1 (Reference)	16	1 (Reference)
Medium	96	16	0.66 (0.30, 1.45)	20	1.51 (0.70, 3.26)	26	1.37 (0.66, 2.85)	22	1.39 (0.65, 2.97)
High	95	23	1.21 (0.57, 2.58)	20	1.22 (0.56, 2.65)	15	0.61 (0.27, 1.38)	20	1.17 (0.54, 2.53)
Continuous log-scale	287	58	0.79 (0.40, 1.54)	58	0.77 (0.41, 1.43)	58	0.76 (0.47, 1.24)	58	1.26 (0.66, 2.40)
Pooled sample									
Continuous log-scale	938	153	1.11 (0.74, 1.66)	153	1.39 (0.93, 2.07)	153	0.99 (0.73, 1.33)	153	1.53 (1.08, 2.15)

CI confidence intervals, N number of women, OR odds ratio, PFAS perfluoroalkyl substances.

The OR analyses are adjusted for parity, gestational week of blood sampling, smoking status, maternal age and BMI.

In addition the pooled analysis is adjusted for country.

FRs are presented in country-specific tertiles and according to a continuous logarithm-transformed scale.

#### PFOS

Higher PFOS levels were associated with slightly reduced FRs in women from Greenland (continuous log-scale FR = 0.83, 95% CI 0.64-1.07) (Table 3) and increased odds for infertility in each of the three countries (ORs of 1.82, 1.92 and 1.22 for high versus low PFOS in women from Greenland, Poland and Ukraine, respectively) (Table 4). However, none of these associations were statistically significant and in the pooled sample there was no strong support for a dose–response relationship between higher PFOS levels and a reduced FR (continuous log-scale FR = 0.90, 95% CI 0.76-1.07) or an increased infertility OR (continuous log-scale OR = 1.39, 95% CI 0.93-2.07).

#### PFHxS

No association was found between levels of PFHxS and FRs (Table 3) or infertility ORs (Table 4) in women from Greenland, Poland or Ukraine or in the pooled sample (continuous log-scale FR = 0.97, 95% CI 0.85-1.11; OR = 0.99, 95% CI 0.73-1.33).

#### PFNA

Women from Greenland with higher levels of PFNA had reduced FRs (medium PFNA: FR = 0.76, 95% CI 0.58-1.00; high PFNA: FR = 0.71, 95% CI 0.54-0.94) compared to women with low levels of PFNA (Table 3). Although not statistically significant, women from Ukraine with medium (FR = 0.79, 95% CI 0.55-1.12) and high levels (FR = 0.88, 95% CI 0.62-1.26) of PFNA also had reduced FRs. In the pooled sample, women with higher PFNA levels had significantly reduced FRs (continuous log-scale FR = 0.80, 95% CI 0.69-0.94) (Table 3). Higher PFNA levels were also associated with increased odds for infertility in women from Greenland (medium PFNA: OR = 1.95, 95% CI 0.95-3.99; high PFNA: OR = 2.15, 95% CI 1.05-4.42) and in the pooled sample (continuous log-scale OR = 1.53, 95% CI 1.08-2.15) (Table 4). In contrast, we found no association between PFNA and FRs or infertility ORs in women from Poland.

### Supplementary findings

In a sensitivity analysis restricted to primiparous women (59% of the women were primiparous), the association between PFNA and the FR in Greenland became weaker (continuous log-scale FR = 0.87, 95% CI 0.60-1.28) and in the pooled sample it became null (continuous log-scale FR = 0.99, 95% CI 0.80-1.22). For PFOA we found a statistically significant association between higher levels and increased FRs in women from Ukraine (continuous log-scale FR = 1.46, 95% CI 1.02-2.08) and in the pooled sample (continuous log-scale FR = 1.31, 95% CI 1.03-1.68). For levels of PFOS and PFHxS no associations were found with the FRs for primiparous women (Additional file 1: Table S1). Regarding the OR for infertility in primiparous women none of the four PFAS were associated with an increased risk of infertility in the pooled sample (Additional file 2: Table S2). Furthermore, no country-specific associations were found between PFOA, PFOS, PFHxS and PFNA and infertility ORs in primiparous women. However, these analyses were based on a limited number of cases.

In addition to the female blood samples, we had blood samples from a subset of 401 male spouses. For PFOA, PFOS and PFHxS there was no consistent association between higher exposure levels in men and prolonged TTP (Additional file 3: Table S3). However, for higher levels of PFNA the FRs were reduced in men from Greenland (continuous log-scale FR = 0.70, 95% 0.50-0.99) and Ukraine (continuous log-scale FR = 0.60, 95% CI 0.33-1.10). In contrast, we found that higher levels of PFNA was associated with an increased FR in men from Poland (continuous log-scale FR = 1.48, 95% CI 0.86-2.56).

## Discussion

We studied the association between serum concentrations of the four most abundant PFAS and couple fecundity as measured by time taken to conceive pregnancies The study populations from Greenland, Poland and Ukraine represented populations with varying PFAS exposure levels and pregnancy planning cultures. Women had on average a 20% reduced chance of achieving pregnancy during one month and a 53% increased risk of infertility per unit increase in PFNA exposure on the log-scale. However, a sensitivity analysis of primiparous women did not unambiguously support that higher levels of PFNA are associated with longer TTP. Also, male spouses with higher levels of PFNA had longer couple TTP. PFOS was less consistently associated and PFOA and PFHxS were not associated with TTP.

Four previous studies have assessed the association between female serum levels of PFAS and female fecundity. Two of these studies involved women from Denmark, but found conflicting results. In a study from 2009 on 1240 women, Fei et al. showed that women with exposure levels of PFOS and PFOA above the 25th percentile had longer TTP than women with exposure levels below the 25th percentile [12]. Specifically, the chance of conceiving during one time period was approximately reduced by 30-40% for women with exposures above the lowest quartile of PFOS and PFOA. The study was based on blood samples and TTP information collected during pregnancy. In contrast, in 2012 Vestergaard et al. did not find any association between TTP and eight PFAS including PFOA, PFOS, PFHxS and PFNA in a prospective study based on 222 first pregnancy planners [16]. In that study women were categorized according to PFAS levels above or below the median concentrations. The median concentrations of PFOA (approximately 6 ng/ml) and PFOS (approximately 35 ng/ml) were almost identical in these two Danish studies and at levels higher than those found in the present study in Greenland (PFOA 1.8 ng/ml, PFOS 20.3 ng/ml), Poland (PFOA 2.7 ng/ml, PFOS 8.0 ng/ml), and Ukraine (PFOA 0.9 ng/ml, PFOS 4.9 ng/ml). In a Norwegian study from 2012 on 910 women, Whitworth et al. found increased odds for subfecundity in parous women with high levels of PFOS and PFOA [14], but they found no association for primiparous women. The median concentrations of PFOS and PFOA were 14 ng/ml and 2 ng/ml, respectively, in the Norwegian study. In an American study from 2013 on 501 couples, Buck Louis et al. looked at couple fecundity in women from Michigan and Texas in relation to various persistent environmental pollutants including seven PFAS [15]. Generally, exposure levels of PFAS in that study were low compared to other studies, and perfluorooctane sulphonamide (PFOSA) was the only PFAS that was negatively associated with couple fecundity. However, in 90% of the analyzed serum samples the level of PFOSA was below the detection limit.

Pregnancy and breastfeeding are two of the major determinants of female PFAS serum concentrations. Maternal PFAS levels are reduced during pregnancy because of the dilution effect of an increased blood volume, and levels are inversely associated with the duration of breastfeeding because of transfer from mother to child [20, 21]. In parous women, the serum PFAS concentration increases with the time since the last pregnancy and breastfeeding period. Parous women with longer TTP for a current pregnancy may therefore be recorded with higher levels of PFAS than women with shorter inter-pregnancy intervals if the blood sample is collected during pregnancy. Thus, including parous women may introduce reverse causality between increased PFAS levels and prolonged TTP [16]. The large majority of participants from Poland and Ukraine were primiparous, but of the women from Greenland only 118 (31%) were first-time mothers. When we restricted the analyses to these primiparous women the association with PFNA practically disappeared.

Substantial differences were found between the study populations from Greenland, Poland and Ukraine in exposure levels of PFOS with median levels at concentrations four times higher in women from Greenland than from Ukraine. Also, for PFOA we found large exposure differences with women from Poland exposed to almost three times higher levels of PFOA than women from Ukraine. In contrast, we found that exposure levels of PFNA and PFHxS were similar in the three study populations. Thus, the lack of association between PFNA and TTP in women from Poland cannot be explained by lower exposure levels in these women.

TTP is a measure that reflects both the male and female reproductive capability as well as the early survival of the fetus. So, the TTP in itself does not indicate if a delayed conception is caused by male or female factors. Moreover, in Greenland a close correlation was found between levels of PFNA in women and men (r_S_ = 0.52), and couple TTP was similarly associated with female serum levels of PFNA as with male serum levels of PFNA.

Although numerous studies in experimental animals documents toxic effects of PFAS on several organ systems, there are no animal experimental data that directly demonstrates effects on male or female fecundity and possible mechanisms remain speculative [7, 8]. Also, it is not clear how PFAS might interfere with human fecundity For instance disturbance of the menstrual cycle [22] and risk of miscarriage [23, 24] is not clearly related to PFAS exposure. On the other hand there is limited evidence that some PFAS are associated with reduced semen quality [19]. This is the first study to examine TTP in relation to both male and female PFAS exposure.

The study has a number of limitations that need consideration. The time window of interest is from the time of the first pregnancy attempt onwards, and our measurement of PFAS levels during pregnancy may not be representative for this period. Especially the transplacental transfer from mother to child and dilution because of increased blood volume seem to impact the serum concentrations of PFAS in pregnancy [6]. We adjusted for the gestational week of blood sampling, but still we cannot exclude the possibility of non-differential misclassification especially in women from Poland who had blood samples collected late in pregnancy (median collection time being gestational week 33). Furthermore, women who failed to conceive or had an early miscarriage were not included in the study. Delayed time to pregnancy because of miscarriage caused by PFAS exposure would not be detected. Only women who plan or partly plan a pregnancy can report a TTP and those who become pregnant despite use of birth control have no eligible TTP value.

Couple fecundity is the chance to conceive during one menstrual cycle, and group-based TTP information from a large sample of couples might provide a good approximation of fecundity in relevant groups of interest. However, several determinants are important for the comparability of TTP and couple fecundity. Couples have to stop using birth control and start attempting pregnancy at an exact time-point with regular unprotected intercourse for the TTP to be a valid measure of fecundity. Obviously, cultural and possibly behavioural differences regarding pregnancy planning exist between the three study populations [17] that might affect this approximation of the TTP as a measure of couple fecundity. Nevertheless, this study does not support a role of PFOA, PFOS and PFHxS on human fecundity. For PFNA we found that women with higher levels took longer time to achieve pregnancy and had an increased risk of infertility than women with lower exposure levels. However, this association was not consistently found in all three study populations despite similar exposure levels, and generally the analysis of primiparous women did not support an association between PFAS and TTP.

## Electronic supplementary material

Additional file 1: Table S1: Fecundability ratios in primiparous women according to female serum concentrations of PFAS. FRs are presented overall and specifically for Greenland, Poland and Ukraine. The tertiles are based on the PFAS limits in the main analysis. (DOC 50 KB)

Additional file 2: Table S2: Infertility odds ratios according to female serum concentrations of PFAS in primiparous women. FRs are presented in country-specific tertiles and according to a continuous logarithm-transformed scale. (DOC 57 KB)

Additional file 3: Table S3: Fecundability ratios according to male serum concentrations of PFAS. FRs are presented in country-specific tertiles and according to a continuous logarithm-transformed scale. (DOC 45 KB)
