# Supplementary material for: Perfluoroalkyl substances and time to pregnancy in couples from Greenland, Poland and Ukraine
Source: Environ Health. 2014 Dec 22;13:116. doi: 10.1186/1476-069X-13-116 (PMC4391306; doi:10.1186/1476-069X-13-116)
Supplement: Supplementary file 1 — Additional file 1: Table S1: Fecundability ratios in primiparous women according to female serum concentrations of PFAS. FRs are presented overall and specifically for Greenland, Poland and Ukraine. The tertiles are based on the PFAS limits in the main analysis. (DOC 50 KB) [file 12940_2014_837_MOESM1_ESM.doc]

| **Supplementary table 1** Fecundability ratios in primiparous women according to female serum concentrations of PFAS. FRs are presented overall and specifically for Greenland, Poland and Ukraine. The tertiles are based on the PFAS limits in the main analysis | | | | | | | | | | | |
| --- | --- | --- | --- | --- | --- | --- | --- | --- | --- | --- | --- |
|  | PFOA | |  | PFOS | |  | PFHxS | |  |  | PFNA |
| Greenland | N | FR (95% CI) |  | N | FR (95% CI) |  | N | FR (95% CI) |  | N | FR (95% CI) |
| Low | 15 | 1 (Reference) |  | 35 | 1 (Reference) |  | 41 | 1 (Reference) |  | 48 | 1 (Reference) |
| Medium | 44 | 1.44 (0.66, 3.12) |  | 55 | 0.67 (0.40, 1.12) |  | 46 | 1.02 (0.60, 1.72) |  | 51 | 0.72 (0.44, 1.18) |
| High | 79 | 1.14 (0.54, 2.40) |  | 48 | 0.96 (0.57, 1.62) |  | 51 | 1.07 (0.63, 1.82) |  | 39 | 0.87 (0.52, 1.45) |
| Continuous log-scale | 138 | 1.09 (0.58, 2.02) |  | 138 | 1.03 (0.64, 1.67) |  | 138 | 1.18 (0.79, 1.77) |  | 138 | 0.87 (0.60, 1.28) |
| Poland |  |  |  |  |  |  |  |  |  |  |  |
| Low | 59 | 1 (Reference) |  | 59 | 1 (Reference) |  | 59 | 1 (Reference) |  | 63 | 1 (Reference) |
| Medium | 66 | 0.92 (0.60, 1.40) |  | 64 | 0.96 (0.61, 1.49) |  | 65 | 0.99 (0.64, 1.52) |  | 64 | 1.01 (0.66, 1.55) |
| High | 62 | 1.09 (0.70, 1.70) |  | 64 | 1.02 (0.67, 1.58) |  | 63 | 1.02 (0.66, 1.56) |  | 60 | 1.40 (0.91, 2.14) |
| Continuous log-scale | 187 | 1.24 (0.79, 1.95) |  | 187 | 1.04 (0.64, 1.68) |  | 187 | 0.97 (0.69, 1.36) |  | 187 | 1.40 (0.95, 2.06) |
| Ukraine |  |  |  |  |  |  |  |  |  |  |  |
| Low | 73 | 1 (Reference) |  | 74 | 1 (Reference) |  | 75 | 1 (Reference) |  | 79 | 1 (Reference) |
| Medium | 77 | 1.34 (0.90, 1.99) |  | 82 | 0.91 (0.61, 1.35) |  | 77 | 0.83 (0.55, 1.26) |  | 82 | 0.76 (0.51, 1.12) |
| High | 77 | 1.20 (0.79, 1.82) |  | 71 | 1.02 (0.68, 1.54) |  | 75 | 1.34 (0.90, 1.99) |  | 66 | 0.98 (0.66, 1.47) |
| Continuous log-scale | 227 | 1.46 (1.02, 2.08) |  | 227 | 1.19 (0.85, 1.67) |  | 227 | 1.18 (0.92, 1.50) |  | 227 | 0.86 (0.60, 1.23) |
| Pooled sample |  |  |  |  |  |  |  |  |  |  |  |
| Continuous log-scale | 552 | 1.31 (1.03, 1.68) |  | 552 | 1.09 (0.86, 1.37) |  | 552 | 1.12 (0.94, 1.33) |  | 552 | 0.99 (0.80, 1.22) |
| CI confidence intervals, FR fecundability ratio, N number of women, PFAS perfluoroalkyl substances | | | | | | | | | | | |
| The FR analyses are adjusted for gestational week of blood sampling, smoking status, maternal age and BMI. In addition the overall analysis is adjusted for country | | | | | | | | | | | |
