# Supplementary material for: Perfluoroalkyl substances and time to pregnancy in couples from Greenland, Poland and Ukraine
Source: Environ Health. 2014 Dec 22;13:116. doi: 10.1186/1476-069X-13-116 (PMC4391306; doi:10.1186/1476-069X-13-116)
Supplement: Supplementary file 2 — Additional file 2: Table S2: Infertility odds ratios according to female serum concentrations of PFAS in primiparous women. FRs are presented in country-specific tertiles and according to a continuous logarithm-transformed scale. (DOC 57 KB) [file 12940_2014_837_MOESM2_ESM.doc]

| **Supplementary table 2** Infertility odds ratios according to female serum concentrations of PFAS in primiparous women. FRs are presented in country-specific tertiles and according to a continuous logarithm-transformed scale | | | | | | | | | | | | | | | |
| --- | --- | --- | --- | --- | --- | --- | --- | --- | --- | --- | --- | --- | --- | --- | --- |
|  | N | n | PFOA |  | N | n | PFOS |  | N | n | PFHxS |  | N | n | PFNA |
| Greenland |  |  | OR (95% CI) |  |  |  | OR (95% CI) |  |  |  | OR (95% CI) |  |  |  | OR (95% CI) |
| Low | 15 | 3 | 1 (Reference) |  | 35 | 2 | 1 (Reference) |  | 41 | 6 | 1 (Reference) |  | 48 | 4 | 1 (Reference) |
| Medium | 44 | 4 | 0.73 (0.14, 3.78) |  | 55 | 10 | 2.39 (0.54, 10.5) |  | 46 | 5 | 0.74 (0.19, 2.89) |  | 51 | 7 | 1.27 (0.33, 4.86) |
| High | 79 | 9 | 0.82 (0.18, 3.77) |  | 48 | 4 | 1.24 (0.25, 6.18) |  | 51 | 5 | 0.58 (0.14, 2.42) |  | 39 | 5 | 1.05 (0.26, 4.25) |
| Continuous log-scale | 138 | 16 | 0.71 (0.17, 3.01) |  | 138 | 16 | 1.11 (0.30, 4.04) |  | 138 | 16 | 0.47 (0.15, 1.51) |  | 138 | 16 | 0.96 (0.33, 2.74) |
| Poland |  |  |  |  |  |  |  |  |  |  |  |  |  |  |  |
| Low | 59 | 8 | 1 (Reference) |  | 59 | 7 | 1 (Reference) |  | 59 | 9 | 1 (Reference) |  | 63 | 13 | 1 (Reference) |
| Medium | 66 | 12 | 1.40 (0.53, 3.66) |  | 64 | 12 | 1.51 (0.55, 4.17) |  | 65 | 11 | 1.16 (0.44, 3.06) |  | 64 | 10 | 0.88 (0.35, 2.21) |
| High | 62 | 10 | 1.54 (0.56, 4.20) |  | 64 | 11 | 1.41 (0.51, 3.84) |  | 63 | 10 | 1.02 (0.38, 2.74) |  | 60 | 7 | 0.58 (0.22, 1.55) |
| Continuous log-scale | 187 | 30 | 1.09 (0.41, 2.91) |  | 187 | 30 | 1.68 (0.55, 5.14) |  | 187 | 30 | 1.02 (0.47, 2.20) |  | 187 | 30 | 0.66 (0.27, 1.61) |
| Ukraine |  |  |  |  |  |  |  |  |  |  |  |  |  |  |  |
| Low | 73 | 14 | 1 (Reference) |  | 74 | 11 | 1 (Reference) |  | 75 | 13 | 1 (Reference) |  | 79 | 12 | 1 (Reference) |
| Medium | 77 | 10 | 0.50 (0.20, 1.24) |  | 82 | 18 | 1.67 (0.72, 3.88) |  | 77 | 20 | 1.52 (0.67, 3.44) |  | 82 | 18 | 1.51 (0.66, 3.46) |
| High | 77 | 16 | 1.01 (0.44, 2.34) |  | 71 | 11 | 1.24 (0.49, 3.13) |  | 75 | 7 | 0.51 (0.19, 1.34) |  | 66 | 10 | 1.01 (0.41, 2.50) |
| Continuous log-scale | 227 | 40 | 0.59 (0.27, 1.25) |  | 227 | 40 | 0.74 (0.37, 1.49) |  | 227 | 40 | 0.65 (0.37, 1.14) |  | 227 | 40 | 1.15 (0.56, 2.34) |
| Pooled sample |  |  |  |  |  |  |  |  |  |  |  |  |  |  |  |
| Continuous log-scale | 552 | 86 | 0.70 (0.40, 1.21) |  | 552 | 86 | 0.91 (0.53, 1.55) |  | 552 | 86 | 0.72 (0.48, 1.08) |  | 552 | 86 | 0.93 (0.57, 1.51) |
| CI confidence intervals, N total number of women, n number of women with time to pregnancy >13 months, OR odds ratio, PFAS perfluoroalkyl substances | | | | | | | | | | | | | | | |
| The OR analyses are adjusted for gestational week of blood sampling, smoking status, maternal age and BMI. In addition the pooled analysis is adjusted for country | | | | | | | | | | | | | | | |
