# Supplementary material for: Perfluoroalkyl substances and time to pregnancy in couples from Greenland, Poland and Ukraine
Source: Environ Health. 2014 Dec 22;13:116. doi: 10.1186/1476-069X-13-116 (PMC4391306; doi:10.1186/1476-069X-13-116)
Supplement: Supplementary file 3 — Additional file 3: Table S3: Fecundability ratios according to male serum concentrations of PFAS. FRs are presented in country-specific tertiles and according to a continuous logarithm-transformed scale. (DOC 45 KB) [file 12940_2014_837_MOESM3_ESM.doc]

| **Supplementary table 3** Fecundability ratios according to male serum concentrations of PFAS. FRs are presented in country-specific tertiles and according to a continuous logarithm-transformed scale | | | | | | | | |
| --- | --- | --- | --- | --- | --- | --- | --- | --- |
|  | N | PFOA |  | PFOS |  | PFHxS |  | PFNA |
| Greenland |  | FR (95% CI) |  | FR (95% CI) |  | FR (95% CI) |  | FR (95% CI) |
| Low | 54 | 1 (Reference) |  | 1 (Reference) |  | 1 (Reference) |  | 1 (Reference) |
| Medium | 53 | 1.18 (0.73, 1.91) |  | 0.77 (0.48, 1.24) |  | 0.77 (0.48, 1.21) |  | 0.62 (0.39, 1.00) |
| High | 53 | 0.95 (0.59, 1.54) |  | 0.72 (0.44, 1.18) |  | 0.93 (0.57, 1.53) |  | 0.81 (0.50, 1.32) |
| Continuous log-scale | 160 | 1.13 (0.62, 2.04) |  | 0.71 (0.44, 1.15) |  | 0.84 (0.58, 1.23) |  | 0.70 (0.50, 0.99) |
| Poland |  |  |  |  |  |  |  |  |
| Low | 49 | 1 (Reference) |  | 1 (Reference) |  | 1 (Reference) |  | 1 (Reference) |
| Medium | 49 | 0.71 (0.43, 1.18) |  | 1.01 (0.63, 1.64) |  | 0.85 (0.51, 1.40) |  | 1.13 (0.70, 1.85) |
| High | 48 | 0.81 (0.49, 1.35) |  | 0.99 (0.60, 1.64) |  | 1.67 (1.00, 2.77) |  | 1.06 (0.65, 1.72) |
| Continuous log-scale | 146 | 0.84 (0.46, 1.52) |  | 1.12 (0.57, 2.19) |  | 1.86 (0.97, 3.57) |  | 1.48 (0.86, 2.56) |
| Ukraine |  |  |  |  |  |  |  |  |
| Low | 32 | 1 (Reference) |  | 1 (Reference) |  | 1 (Reference) |  | 1 (Reference) |
| Medium | 32 | 1.59 (0.83, 3.08) |  | 1.72 (0.89, 3.32) |  | 1.16 (0.60, 2.23) |  | 0.83 (0.43, 1.61) |
| High | 31 | 0.95 (0.49, 1.84) |  | 1.12 (0.59, 2.12) |  | 1.02 (0.54, 1.93) |  | 0.72 (0.38, 1.39) |
| Continuous log-scale | 95 | 0.92 (0.61, 1.38) |  | 1.01 (0.59, 1.74) |  | 0.99 (0.55, 1.76) |  | 0.60 (0.33, 1.10) |
| Pooled sample |  |  |  |  |  |  |  |  |
| Continuous log-scale | 401 | 0.99 (0.75, 1.30) |  | 0.90 (0.66, 1.22) |  | 1.00 (0.77, 1.30) |  | 0.81 (0.63, 1.04) |
| CI confidence intervals, FR fecundability ratio, N number of men, PFAS perfluoroalkyl substances | | | | | | | | |
| The FR analyses are adjusted for paternal and maternal age and male BMI. In addition the pooled analysis is adjusted for country | | | | | | | | |
